# Inflammatory status in pediatric sickle cell disease: Unravelling the role of immune cell subsets

**DOI:** 10.3389/fmolb.2022.1075686

**Published:** 2023-01-10

**Authors:** Silvio Marchesani, Valentina Bertaina, Olivia Marini, Matilde Cossutta, Margherita Di Mauro, Gioacchino Andrea Rotulo, Paolo Palma, Letizia Sabatini, Maria Isabella Petrone, Giacomo Frati, Giulia Monteleone, Giuseppe Palumbo, Giulia Ceglie

**Affiliations:** ^1^ University Department of Pediatrics, Bambino Gesù Children’s Hospital, University of Rome Tor Vergata, Rome, Italy; ^2^ Department of Pediatric Hematology and Oncology, Cell and Gene Therapy, Bambino Gesù Children’s Hospital, IRCCS, Rome, Italy; ^3^ Women’s and Children’s Health Department, Hematology-Oncology Clinic and Laboratory, University of Padova, Padova, Italy; ^4^ Clinical and Research Unit of Clinical Immunology and Vaccinology, Academic Department of Pediatrics (DPUO), Bambino Gesù Children Hospital, IRCCS, Rome, Italy; ^5^ Department of Neuroscience, Rehabilitation Ophthalmology Genetics Maternal and Child Health (DINOGMI), University of Genoa, Genoa, Italy; ^6^ Department of Systems Medicine, University of Rome Tor Vergata, Rome, Italy

**Keywords:** sickle cell disease, immune system, immunophenotype, anemia, hemoglobinopathies, flow cytofluorimetry

## Abstract

**Introduction:** The mutation of the beta-globin gene that causes sickle cell disease (SCD) results in pleiotropic effects, such as hemolysis and vaso-occlusive crisis that can induce inflammatory mechanisms with deleterious consequences on the organism. Moreover, SCD patients display an increased susceptibility to infections. Few studies are currently available that evaluate a wide immunological profile in a pediatric population. This study proposes an evaluation of the immune profile in subjects with SCD in a pediatric population through a detailed analysis by flow cytometry.

**Methods and Materials:** Peripheral blood samples from 53 pediatric patients with SCD (mean age 9.8 years, interquartile range 9 years) were obtained and then analyzed by flow cytometry, in order to evaluate changes in the immune populations compared to 40 healthy donors (mean age 7.3 years, interquartile range 9.5 years).

**Results:** Our data showed an increase in neutrophils (with a reduction in the CD62L + subpopulation) and monocytes (with a decrease in HLA-DRlow monocytes) with normal values of lymphocytes in SCD patients. In the lymphocyte subpopulations analysis we observed lower values of CD4^+^ T cells (with higher number of memory and central memory T lymphocytes) with increased frequency of CD8^+^ T cells (with a predominant naive pattern). Moreover, we observed higher values of CD39^+^ Tregs and lower HLA-DR+ and CD39^−^ T cells with an increased Th17, Th1-17 and Th2 response.

**Conclusion:** We observed immunological alterations typical of an inflammatory status (increase in activated neutrophils and monocytes) associated with a peculiar Treg pattern (probably linked to a body attempt to minimize inflammation intrinsic to SCD). Furthermore, we highlighted a T helper pathway associated with inflammation in line with other studies. Our data showed that immunological markers may have an important role in the understanding the pathophysiology of SCD and in optimizing targeted therapeutic strategies for each patient.

## 1 Introduction

Sickle cell disease (SCD) is a genetic disease caused by a single mutation in the beta-globin gene (Pinto et al., 2019). The resulting modified gene determines the production of an abnormal hemoglobin called hemoglobin S (HbS) which tends to polymerize under de-oxygenating conditions (Lonergan et al., 2001): this changes the rheology of red blood cells (RBCs) with a consequent drastic reduction of their half-life and multiple cascade effects including anemia, frequent vaso-occlusive crises (VOC), painful crises, strokes, multi-organ dysfunction and premature death (Ware et al., 2017).

It is widely described in current literature how these changes in the red blood cells’ structure induce pro-inflammatory effects with deleterious consequences, giving SCD the characteristics of a chronic inflammatory condition (Conran and Belcher, 2018).

In fact, innate immune cells (monocytes, dendritic cells, neutrophils, eosinophils, basophils, natural killer (NK) cells, invariant natural killer (iNK) T cells and platelets, along with tissue-resident macrophages and mast cells) have been linked to an increasingly important role in promoting inflammation, adhesion and Vaso-Occlusive painful crisis in SCD (Conran and Belcher, 2018).

Multiple studies highlighted that free hemoglobin and heme released by hemolysis can play a crucial role in the inflammatory process through the activation of toll-like receptor (TLR) 4 or through the generation of reactive species of oxygen, with consequent activation of the inflammasome (Cerqueira et al., 2011; Belcher et al., 2014; Paul et al., 2014). In addition, the depletion of nitric oxide by free hemoglobin, with activation of platelets and mast cells may contribute to this process (Dutra et al., 2014). Moreover, ischemia-reperfusion damage following vaso-occlusive (Kalogeris et al., 2012) is associated with the release of DAMPs, such as ATP, heme, and heat shock proteins (Adewoye et al., 2005 ; Xu et al., 2014) with a consequent exacerbation of inflammatory mechanism (Idzko et al., 2014). In addition, other inflammatory pathways were described (Conran and Belcher, 2018), such as the formation of thrombin (Whelihan et al., 2016 ; Wun and Brunson, 2016) and the activation of the alternative complement pathway(Chudwin et al., 1994), as well as increased susceptibility to infections (Nickel and Hsu, 2016).

Innate immune cells possess a large repertoire of receptors, including receptors for cytokines and chemokines, which enable them to respond to various inflammatory signals and the activation of innate immune cells in SCD is probably promoted by both chronic and acute inflammation (Keikhaei et al., 2013). Once activated, innate immune cells release a wide range of cytokines and chemokines, thus promoting a vicious cycle of immune cell recruitment and activation (Lanaro et al., 2009; Qari et al., 2012). Pro-inflammatory cytokines, produced mainly by monocytes and macrophages but also by platelets and other innate immune cells, can activate the endothelium along with heme, resulting in increased expression of adhesion molecules (Pathare et al., 2004; Sakamoto et al., 2013). Neutrophils are crucial factors in endothelial adhesion, but monocytes and platelets also contribute to decreased blood flow and vaso-occlusion through the formation of red blood cells (RBC)-monocytes, platelets-monocytes and platelets-neutrophils aggregates (Zhang et al., 2016).

Moreover, patients with SCD are predisposed to infections mainly due to splenic dysfunction which can favor deficits in innate, humoral and cellular immune function (Ochocinski et al., 2020).

There are currently few studies (Rautonen et al., 1992; Balandya et al., 2016; Giulietti et al., 2022) in the literature that evaluate a large number of immune system cells in pediatric patients with SCD. This study proposes an evaluation of the immune profile in subjects with sickle cell disease in a pediatric population through a detailed analysis by flow cytometry, in order to better understand the involvement of the immune system in the pathophysiology of this disease in pediatric age.

## 2 Methods and materials

### 2.1 Population study

We studied a cohort of patients currently in follow-up at our center (Ospedale Pediatrico Bambino Gesù in Rome) for SCD at steady state (no current hemolytic crises or vaso-occlusive crises in the last month or other acute complications at the time of the blood test), medical information were collected by revising their clinical records.

As control group, healthy donors (HD) were selected. We selected as HD patients who came to our clinic to perform screening blood tests that comprised immunephenotyping analysis.

Both groups were matched for age and ethnicity and informed consent was obtained from all legal guardians.

Regarding inclusion criteria, none of the patients or HD.

• Had history of major infectious episodes in the 12 months prior blood collection, with hospitalization or need for intravenous/intramuscular antibiotic therapy;
• Presented allergic or autoimmune diseases;
• Underwent therapies which can modulate the immune system;
• Received vaccinations in the 4 weeks prior the blood collections;
• Had ongoing VOC or hemolytic crisis at the moment of the blood test;
• Was pregnant;
• Had other chronic diseases or infections.

Afterwards, we divided patients considering spleen alterations (patients with normal spleen function *versus* patients who underwent splenectomy or experienced auto-splenectomy), number of vaso-occlusive crisis (VOC, defined as a crisis characterized by pain related to SCD’s pathophysiology which requires hospitalization) and number of transfusion procedures.

At the moment, we are not aware of studies in literature that support a standardized subdivision of patients according to number of VOC and number of transfusion procedures, consequently, based on our experience, we divided the patiets as follows: for VOC, our population was divided in patients who experienced 1 or more crisis per year *versus* less than 1 crisis per year; as for the number of transfusions, the two subgroups considered were patients who underwent 2 or more transfusions per year *versus* less than 2 transfusions per year.

### 2.2 Study assay

We examined complete blood count lymphocytes and myeloid subpopulations using flow cytometry.

The immunophenotype was examined by multiparametric flow cytometry. Patients’ peripheral venous blood was collected in test tubes containing ethylenediaminetetraacetic acid (EDTA) as anticoagulant and analyzed within 24 h from collection to determine the percentage of different white blood cell subpopulations. For each sample, 120 µL of blood were incubated for 15 min at room temperature in the dark in the presence of monoclonal antibodies. Subsequently each sample was subjected to osmotic lysis to eliminate contaminating red blood cells using 2 ml of commercial ammonium chloride solution (150 mM NH4Cl, 10 mM NaHCO3 pH 7·4, BD Pharma Lyse TM, BD Biosciences, San Diego, CA, United States) for 10 min at room temperature. The labeled cell suspension was then centrifuged at 400 g for 5 min at room temperature to separate the lysed red blood cell supernatant from the labeled white blood cells. The supernatant was then removed and the labeled cell pellet resuspended in 200 µl of Phosphate Saline Buffer (PBS) to be immediately acquired by DxFlex Beckman Coulter Flow Cytometer (Milano, Italy).

To reach enough cells analyzed and to obtain statistical significance, at least 150,000 events/cells were recorded for each sample. Immunophenotypic analysis to determine white blood cell subpopulations was performed using Beckman Coulter DxFlex Flow Cytometer and analyzed using FlowJo Software (Becton Dickinson and Company, United States) according to different analysis strategies. Briefly, anti-CD3, anti-CD4, anti-CD8, anti-CD19, anti-CD16/CD56 monoclonal antibodies were used to discriminate T and B lymphocytes and NK cells. Anti-Vα24JαQ TCR Chain was used to identify iNKT cells.

T cells were also further characterized into CD25^+^CD127low T regulatory CD4^+^ cells (Tregs). Anti-HLA-DR was used to discriminate activated T regs (CD4^+^ CD25^+^CD127low HLA-DR+) and non-activated Tregs (CD4^+^ CD25^+^CD127low HLA-DR-). Moreover we have identified CCR7+CD45RA + naïve, CCR7-CD45RA-effector memory, CCR7+CD45RA-central memory and CCR7-CD45RA + effector memory RA T cell subpopulations. Anti-CD183 (CXCR3), Anti-CD196 (CCR6), anti-CD194 (CCR4), and anti-CCR10 were used to identify T helper Th1 (CCR4-CXCR3+CCR10-CCR6-), Th2 (CCR4+CXCR3-CCR10-CCR6-), Th17 (CCR4+CXCR3-CCR10-CCR6+), Th17/Th1 (CCR4+CXCR3-CCR10-CCR6+), Th22 (CCR4+CCR6+CCR10 + CXCR3-). Anti -CD69 was used to identify activated CD4^+^ and CD8^+^ T cells.

For the myeloid cell discrimination, anti-CD33, anti-CD14, anti-CD16 and anti-CD66b monoclonal antibodies were used to distinguish between monocytes and neutrophils. Monocytes were gated according to their forward and side scatter profile and based on their CD33 cell membrane expression, then they were further characterized due to their CD14 and CD16 surface expression. Monocyte subpopulations CD14brightCD16-were classified “classical” (cM), CD14brightCD16+ “intermediate” (iM) and CD14^+^CD16^+^ “non-classical” (ncM), as previously described (Mimiola et al., 2014; Maximova et al., 2019). Among total CD66b+ granulocytes, neutrophils were differentiated from eosinophils according to their forward and side scatter profile and based on their CD16 cell membrane expression. Then specifically on the neutrophil subset, the CD62L, CD35, CD11b, CD62P cell membrane expression was determined as median fluorescence intensity, in order to evaluate the differential cell activation status.

Anti-CD3, anti-CD4, anti-CD8, anti-CD19, anti-CD16, anti-iNKT, anti-CD69, anti-HLA-DR, anti-CD56, anti-CD194, anti-CD196 and anti-CD33, anti-CD35, anti CD62P antibodies were purchased from Becton-Dickinson (Heidelberg, Germany), anti-CD25, anti-CD127, anti-CD45RA, anti-CCR7, anti-CD14, anti-CD45RA, anti-CD45RO, anti-CCR7, anti-CD62L and anti-CD183, anti-CD62L antibodies were purchased from Miltenyi Biotec (Bergisch Gladbach, Germany), while anti-CD66b was purchased from Biolegend (San Diego, CA), anti-CCR10 were purchased from R&D System (Minneapolis, United States).

The list of the aforementioned antibodies is showed in Table 1.

**TABLE 1:** List of antibodies used for flow cytometry in this study.

anti-CD19	anti-CD8	anti-CD45RO	anti-CD127	anti-CD183	anti-CCR10	anti-CD16/CD56	anti-CD14	CD35
anti-CD3	anti-CD45	anti-CD62L	anti-CD25	anti-CD196	anti-CD69	Anti-Vα24JαQ TCR Chain	anti-CD16	CD11b
anti-CD4	anti-CD45RA	anti-CCR7	anti-CD39	anti-CD194	anti-HLA-DR	anti-CD33	anti-CD66b	CD62P

### 2.3 Statistical analysis

Statistical analysis was performed to compare and correlate data. The comparison study between two different groups included the use of Student’s t-test (two-sided) for parametric distribution or Mann-Whitney test for non-parametric distribution. Data considered with statistical significance were those with *p*-value less than .05. When the *p*-values resulted less than .05 we associated asterisks with incremental significance levels: *p ≤ .05; **p ≤ .01; ***p ≤ .001. The statistical analysis and the graphic processing of the data was carried out using the GraphPad Prism software, version 5 for Machintosh (GraphPad Software, Inc.).

## 3 Results

### 3.1 Population study

We studied 53 patients with a diagnosis of SCD (31 males and 22 females, mean age 9.8 years, interquartile range 9 years) and 40 HD (21 males and 19 females, mean age 7.3 years, interquartile range 9.5 years). Patients of HD group were younger than SCD (*p* < .05).

The clinical characteristics of our population are shown in Table 2.

**TABLE 2:** Clinical and laboratory characteristics of SCD population.

Clinical and laboratory charachteristics	Value
Number of female patients (%)	22 (41.5)
Number of male patients (%)	31 (58.5)
Mean age (range)	9.8 (1–31)
Number of patients with HbSS genotype (%)	44 (83)
Number of patients with HbSC genotype (%)	4 (7.6%)
Number of patients with HbSThal genotype (%)	5 (9.4%)
Number of patients taking hydroxyurea (%)	42 (79.2)
Number of patients taking chelation therapy (%)	5 (9.4)
Median number of transfusion procedures per year (range)	1.5 (0–12.5)
Number of patients who underwent transfusion procedures with higher frequency (≥2 per year) (%)	23 (43.4)
Median number of VOC per year (range)	0.5 (0–5.5)
Number of patients who experienced VOC with higher frequency (≥1 per year) (%)	16 (30.2)
Number of patients who routinely underwent eritroexchange procedures (%)	11 (20.7)
Number of patients who excperienced ACS in the last 2 years (%)	4 (7.5)
Number of patients who underwent surgical splenectomy or experienced autosplenectomy (%)	10 (18.9)
Median value of Hb (range) g/dL	9.44 (7.48–13.27)
Median value of Ht (range) %	29.4 (23.14–38.37)
Median value of Ret (range)/mm^3^	331300 (113600–654600)
Median value of Ret (range) %	9.86 (2.1–24.63)
Median value of HbS (range) %	59.3 (27.7–81.47)
Median value of HbF (range) %	9 (0.535–28.1)
Median value of total bilirubin (range) mg/dL	1.79 (0.31–11.54)
Median value of ferritin (range) ng/mL	304.6 (38.5–5704.5)

### 3.2 Leukocytes count and subpopulations

Our data showed an increase in the total leukocyte count in SCD patients (8245/mm^3^ vs. 6240/mm^3^ *p*-value≤.05) mainly due to a higher count of granulocytes (3571/mm^3^ vs. 2495/mm^3^, *p*-value *p* ≤ .001) with no differences in lymphocytes count (3010/mm^3^ vs. 2919/mm^3^, *p*-value .587).

**FIGURE 1:**
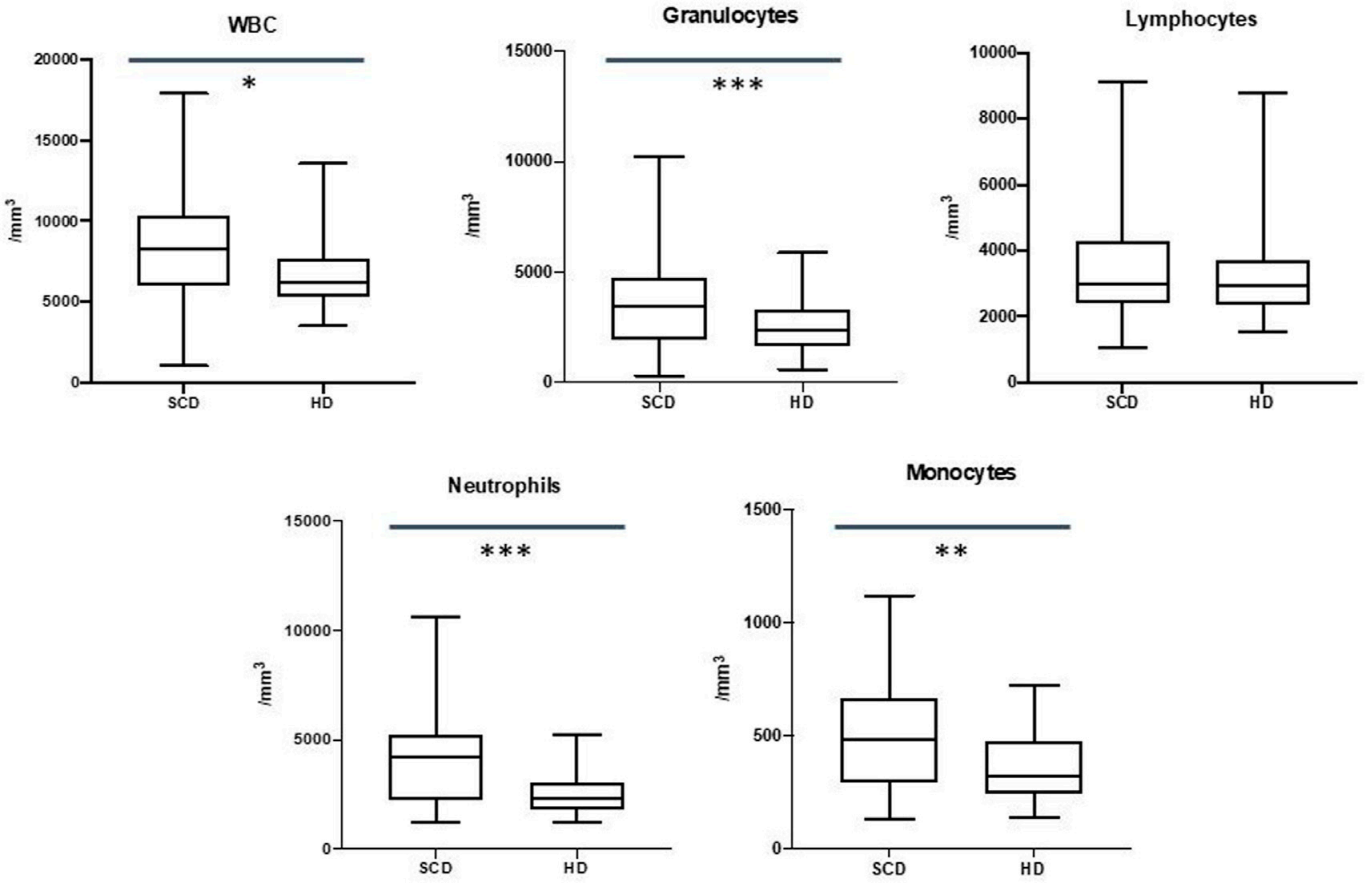
Differences in white blood cells (WBC), granulocytes, lymphocytes, neutrophils and monocytes between sickle cell disease (SCD) patients and healthy donors (HD). *p ≤.05; **p ≤.01; ***p ≤.001.

Patients with SCD displayed an increased count of neutrophils (4225/mm^3^ vs. 2350/mm^3^, *p*-value *p* ≤ .001), monocytes (430/mm^3^ vs 320/mm^3^, *p*-value≤.01) and basophiles (50/mm^3^ vs. 40/mm^3^, *p*-value = 0,0001) in absolute number (Figure 1). We did not observe any differences in the total eosinophil count.

These data are shown in Figure 1.

### 3.3 Neutrophils and monocytes subpopulations

Our data showed a reduced expression of CD62L on neutrophils in SCD patients (310207 Mean Fluorescence Intensity MFI vs. 558018 MFI, *p*-value ≤.01), with no expression variation in CD62P, CD16, CD11b and CD35 markers.

We did not observe differences in monocyte subpopulations, but SCD patients displayed a decrease of HLA-DR^low^ monocytes in the comparison with HD (15.2% vs. 26.1%, *p*-value ≤.05).

**FIGURE 2:**
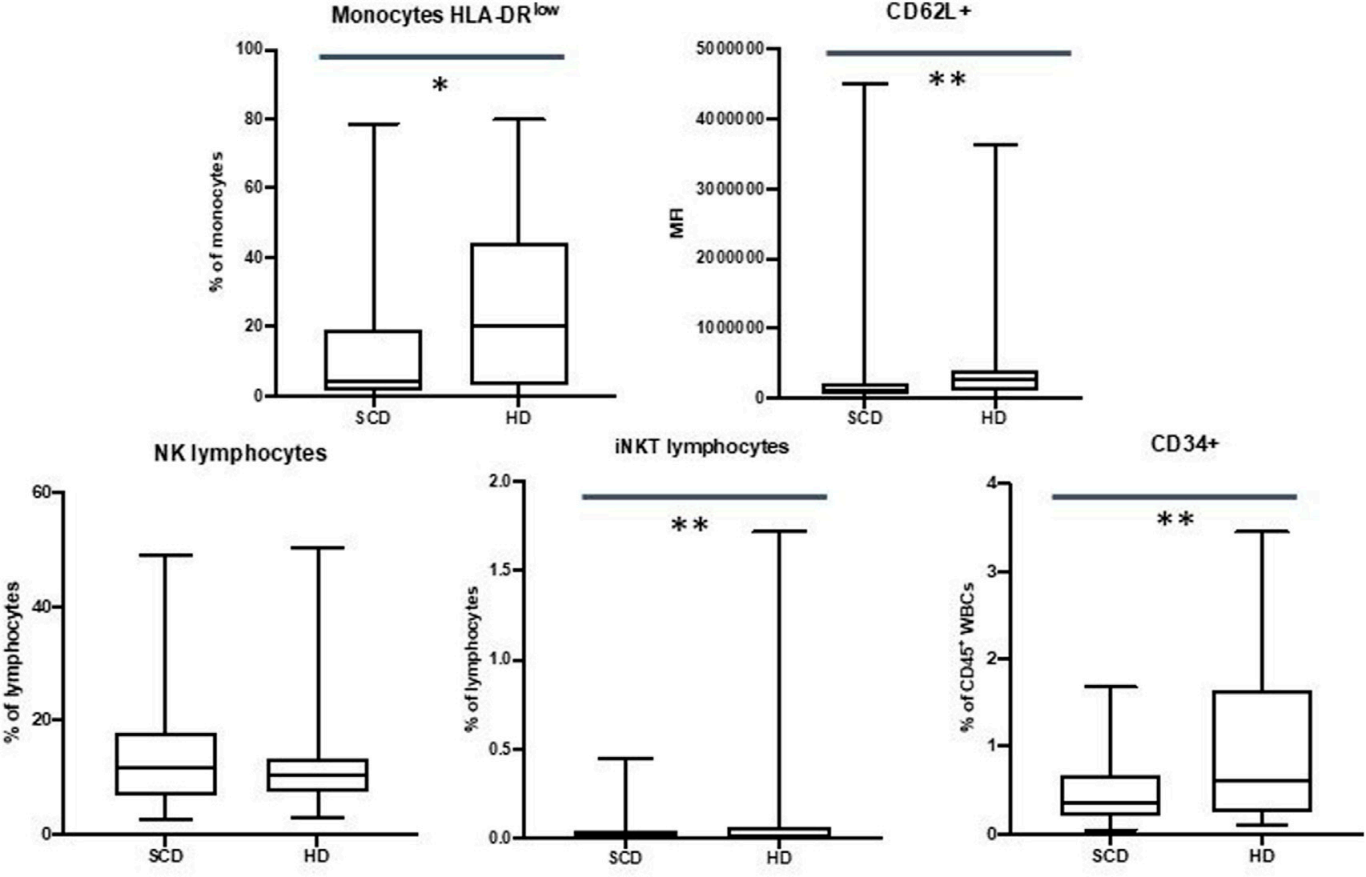
Differences in HLA-DR^low^ monocytes, CD62L + neutrophils, NK lymphocytes, iNKT lymphocytes and CD34^+^ cells between sickle cell disease (SCD) patients and healthy donors (HD). *p ≤.05; **p ≤.01; ***p ≤.001.

These data are shown in Figure 2.

### 3.4 Natural killer cells

We did not observe any differences in NK populations, but data showed a reduction in percentage in iNKT cells in patients with SCD (0.05% vs. 0.25%, *p*-value ≤.01)

These data are shown in Figure 2.

### 3.5 CD34^+^ cells

Our data showed a decrease in CD34^+^ cells in SCD patients when compared to HD (in percentage 0.48% vs. 0.97%, *p*-value ≤.01; in absolute number 40/mm^3^ vs. 61/mm^3^, *p*-value ≤.05).

These data are shown in Figure 2.

### 3.6 Lymphocytes subpopulations

Our data did not show differences in the CD19^+^ subpopulations, but we observed a reduction in the percentage of the CD3^+^ cells in SCD patients (40.9% vs. 49.2%, *p*-value ≤.01).

CD4^+^ T cells were found to be reduced in percentage in SCD patients (19.6% vs. 25.4%, *p*-value ≤.001) with an increase in percentage of CD4^+^ CD45RO + cells (39.6% vs. 31.1%, *p*-value ≤.05) and central memory CD4^+^ T cells (20.6% vs. 12.2%, *p*-value ≤.05). Moreover, we observed an increase in the absolute number of the late effector memory CD45RA+ CD4^+^ T cells (also known as TEMRA: terminally differentiated effector memory cells re-expressing CD45RA) in the SCD cohort (14.4/mm^3^ vs. 18.9/mm^3^, *p*-value ≤.05).

We did not observe differences in the naïve CD4^+^ T cells subpopulations.

Our data highlighted an increase in the CD8^+^ T cells in percentage in SCD patients (25.1% vs. 20.9%, *p*-value ≤.05) with a percentage increase of the CD8^+^ CD45RA + cells (44.6% vs. 21.6%, *p*-value ≤.001).

Patients displayed an increase in CD69^+^ CD4^+^ and CD8^+^ T cells in percentage (for CD4^+^ CD69+: 0.56% vs. 0.27%, *p*-value ≤.01; for CD8^+^ CD69+: 1.5% vs. 0.2%, *p*-value ≤.001) and in absolute number (for CD4^+^ CD69+: 5.9/mm^3^ vs. 3.1/mm^3^, *p*-value ≤.05; for CD8^+^ CD69^+^; 15.6/mm^3^ vs. 2.9/mm^3^, *p*-value ≤.001).

No statistically significant difference regarding the CD4/CD8 ratio between SCD patients and HD was observed (2.2 vs. 2.3, *p*-value = .231).

**FIGURE 3:**
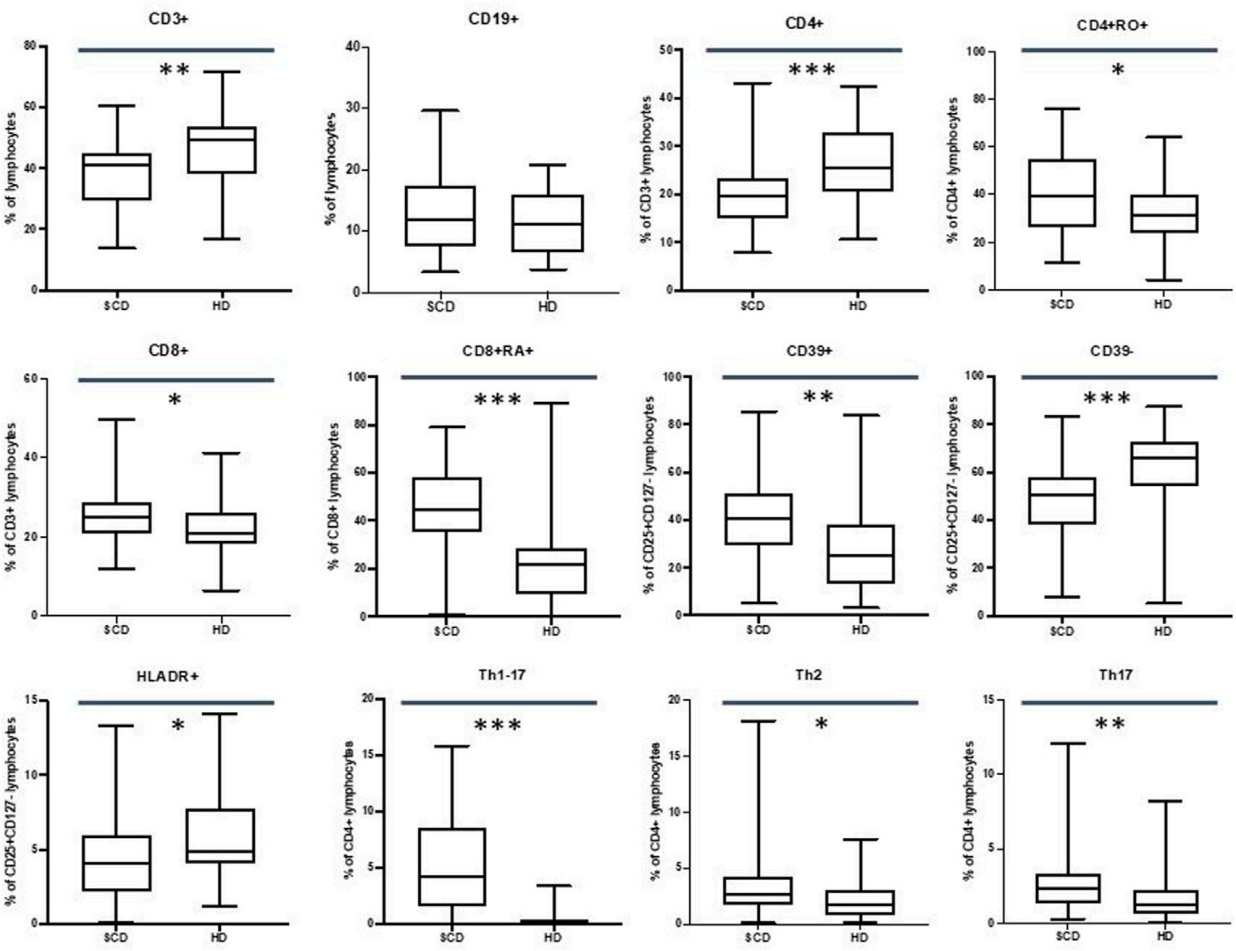
Differences in CD3^+^, CD19^+^, CD4^+^, CD4+RO+, CD8^+^, CD8+RA+, Treg CD39^+^, Treg CD39^−^, Treg HLADR+, Th1-Th17, Th2, Th17 lymphocytes between sickle cell disease (SCD) patients and healthy donors (HD). *p≤.05; **p ≤.01; ***p ≤.001.

These data are shown in Figure 3.

### 3.7 Regulatory T Cells and T helper lymphocytes

Our data showed an increase in CD39^+^ T cells in SCD population in percentage (40.8% vs. 25.3%, *p*-value ≤.01) and in absolute number (21.4/mm^3^ vs. 11.7/mm^3^, *p*-value ≤.001) with a percentage decrease in CD39-T cells (50.7% vs. 65.8%, *p*-value ≤.001).

Moreover, we observed a reduction in the CD4^+^ HLA-DR + population in the patients’ cohort in percentage (4.1% vs. 4.9%, *p*-value ≤.05) and in absolute number (29.6/mm^3^ vs. 40.3/mm^3^, *p*-value ≤.05).

We did not observe any differences in the total Treg CD4^+^ CD25^+^ CD127^low^ population.

Regarding T Helper subpopulations, we highlighted an increase in Th1-Th17 cells in percentage 4.2% vs. 0.2%, *p*-value ≤.001) and in absolute number (22.2/mm^3^ vs. 1.3/mm^3^, *p*-value ≤.001) in SCD population.

Furthermore, patients displayed an increase in percentage of Th2 cells (2.6% vs. 1.7%, *p*-value ≤.05) and Th17 cells (2.4% vs. 1.2% *p*-value ≤.01).

These data are shown in Figure 3.

**FIGURE 4:**
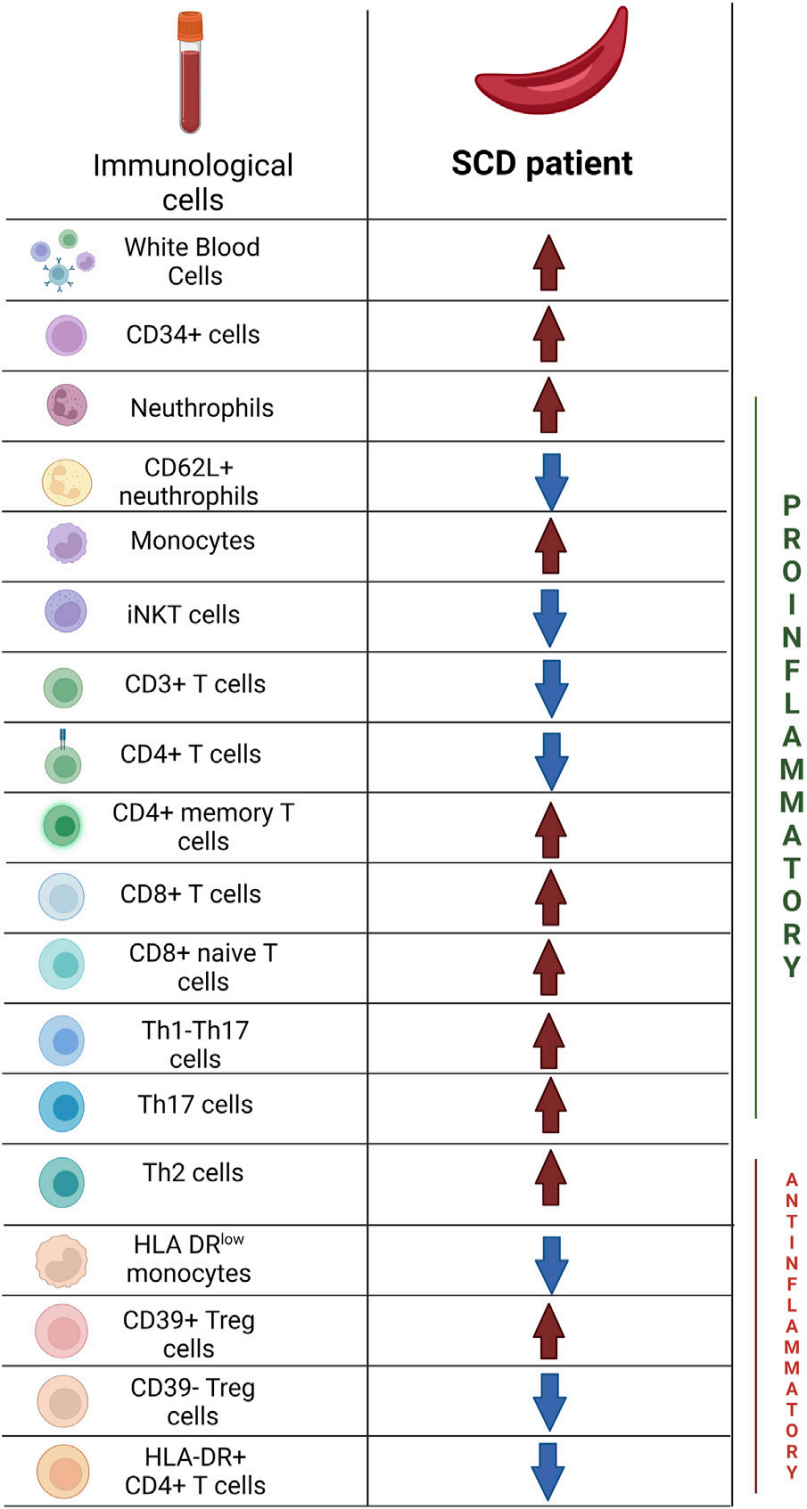
Main differences in immunological profile between Sickle Cell Disease patients and Healthy Donors. Created with BioRender.com.

The aforementioned data are synthesized in Figure 4 and in Tables 3, 4.

**TABLE 3:** Main differences in the immunological profile (in absolute number) between Sickle Cell Disease population and Healthy Donors. *Data with statistical significance. §Data expressed in MFI (Mean Fluorescence Intensity).

Cells	SCD: Median value/mm^3^ (range)	HD: Median value/mm^3^ (range)	*p*-value
White Blood Cells	8245 (1050–17890)	6240 (3500–13540)	.012*
Neutrophils	4225 (1200–10620)	2350 (1250–5220)	.0001*
Lymphocytes	3010 (1060–9140)	2919.5 (1540–8790)	.587
Monocytes	430 (130–1120)	320 (140–720)	.01*
Eosinophils	305 (20–1220)	280 (20–620)	.401
Basophils	50 (10–390)	40 (10–80)	.0001*
CD35^+^ neutrophils	51503 (8458–137870)^§^	56480 (12720–110816)^§^	.45
CD62P + neutrophils	14933 (2960–60881)^§^	14079 (1527–17681)^§^	.283
CD62L + neutrophils	122684 (3266–4500000)^§^	273198 (9342–3630000)^§^	.009*
CD11b + neutrophils	98635 (26603–929000)^§^	89308 (28774–1180000)^§^	.371
HLA-DR^low^ monocytes	15.5 (0–916)	50 (0–596)	.523
CD34^+^ cells	29 (3–178)	37 (5–229)	.044*
CD19^+^ lymphocytes	320.53 (61.38–2310)	337.62 (88.26–1731.63)	.583
CD3^+^ lymphocytes	1144.66 (310.58–4944.74)	1247.33 (414.20–4304.58)	.149
CD4^+^ lymphocytes	630.99 (184.44–2078.39)	692.86 (255.64–2733.99)	.227
CD4^+^ RA + lymphocytes	233.54 (49.47–2037.64)	293.99 (28.66–2343.84)	.236
CD4^+^ RO + lymphocytes	232.32 (42.68–714.27)	207.84 (48.78–829.50)	.31
CD4^+^ central memory lymphocytes	138.49 (0.59–554.91)	88.23 (0.06–874.79)	.057
CD4^+^ effector memory lymphocytes	127.66 (23.16–1013.99)	150.11 (0.78–650.85)	.354
CD4^+^ effector memory RA + lymphocytes	14.40 (0.89–989.68)	18.91 (1.75–445.42)	.047*
CD8^+^ lymphocytes	291.59 (74.22–1353.79)	314.07 (26.51–929.79)	.807
CD8^+^ RA + lymphocytes	118.63 (2.63–959.84)	51.30 (1.89–444.21)	.0001*
CD8^+^ RO + lymphocytes	60.00 (3.23–294.50)	67.52 (3.66–435.66)	.921
CD8^+^ central memory lymphocytes	14.97 (0.10–391.03)	15.58 (0.15–311.71)	.333
CD8^+^ effector memory lymphocytes	74.61 (0.76–394.09)	79.66 (0.33–416.55)	.983
CD8^+^ effector memory RA + lymphocytes	39.32 (0.38–763.54)	50.90 (0.34–290.33)	.053
CD4^+^ CD69^+^ lymphocytes	5.89 (1.05–52.31)	3.11 (0.03–20.98)	.045*
CD8^+^ CD69^+^ lymphocytes	15.57 (1.00–107.86)	2.84 (0.38–18.90)	.0001*
Treg CD4^+^ CD25^+^ CD127- lymphocytes	55.19 (9.34–192.05)	48.52 (3.77–171.76)	.575
Treg CD4^+^ CD39^+^	21.43 (1.06–164.01)	11.75 (2.36–62.75)	.003*
Treg CD4^+^ CD39^−^	24.69 (6.12–95.60)	30.85 (0.20–138.61)	.14
Treg HLADR+	29.58 (0.57–136.54)	40.29 (18.34–99.24)	.02*
Th1 lymphocytes	41.51 (3.32–154.86)	63.75 (14.99–198.46)	.161
Th2 lymphocytes	16.15 (0.63–156.82)	11.91 (0.90–68.92)	.158
Th17 lymphocytes	11.20 (1.67–98.23)	9.70 (0.72–43.77)	.174
Th1-Th17 lymphocytes	22.24 (0.09–152.96)	1.28 (0.05–27.52)	.0001*

That the bold values indicates the data with statistical significance.

**TABLE 4:** Main differences in the immunological profile (in percentage) between Sickle Cell Disease population and Healthy Donors. *Data with statistical significance.

Cells	SCD: Median value % (range)	HD: Median value % (range)	*p*-value
HLA-DR^low^ monocytes	4.1 (0–78.7)	20.2 (0–79.8)	.033*
CD34^+^ cells	0.35 (0.04–1.68)	0.6 (0.1–3.45)	.001*
NK cells	11.65 (2.7–49.1)	10.5 (3.1–50.2)	.344
iNKT cells	0.02 (0–0.45)	0.04 (0–1.72)	.009*
CD19^+^ lymphocytes	11.8 (3.3–29.6)	11.2 (3.74–20.8)	.594
CD3^+^ lymphocytes	40.9 (13.6–60.5)	49.2 (16.8–71.5)	.005*
CD4^+^ lymphocytes	19.60 (7.94–43.10)	25.45 (10.6–42.4)	.0004*
CD4^+^ RA + lymphocytes	38.5 (10.6–67.5)	44.1 (16.1–82.5)	.71
CD4^+^ RO + lymphocytes	39.6 (11.5–76.2)	31.1 (4.25–64)	.024*
CD4^+^ central memory lymphocytes	20.6 (0.06–57.3)	12.25 (0.02–62.8)	.017*
CD4^+^ effector memory lymphocytes	20 (2.5–99.8)	23.8 (0.12–57.6)	.578
CD4^+^ effector memory RA + lymphocytes	2.38 (0.15–50.6)	3.72 (0.15–80.5)	.108
CD8^+^ lymphocytes	25.1 (12–49.5)	20.9 (6.4–41.1)	.015*
CD8^+^ RA + lymphocytes	44.6 (0.92–79)	21.6 (0.48–89)	.0001*
CD8^+^ RO + lymphocytes	23.3 (1.99–72.6)	25.6 (1.28–66.2)	.667
CD8^+^ central memory lymphocytes	4.86 (0.14–81.4)	5.08 (0.04–58.1)	.839
CD8^+^ effector memory lymphocytes	25.2 (0.55–74.6)	32.9 (0.07–94)	.179
CD8^+^ effector memory RA + lymphocytes	18.55 (0.17–62.5)	18 (0.08–84.9)	.953
CD4^+^ CD69^+^ lymphocytes	0.56 (0.06–1.86)	0.27 (0.003–1.73)	.0064*
CD8^+^ CD69^+^ lymphocytes	1.53 (0.07–7.01)	0.20 (0.02–1.66)	.0001*
Treg CD4^+^ CD25^+^ CD127- lymphocytes	7.11 (3.21–11.4)	7.06 (0.52–14.3)	.898
Treg CD4^+^ CD39^+^	40.8 (5.04–85.4)	25.3 (3.28–83.8)	.0007*
Treg CD4^+^ CD39^−^	50.7 (7.84–83)	65.85 (5.41–87.4)	.0001*
Treg HLADR+	4.09 (0.07–13.3)	4.86 (1.19–14.1)	.031*
Th1 lymphocytes	7.73 (0.45–23.6)	7.66 (1.21–20.9)	.949
Th2 lymphocytes	2.60 (0.12–18.1)	1.71 (0.14–7.55)	.015*
Th17 lymphocytes	2.37 (0.27–12.1)	1.21 (0.07–8.24)	.0042*
Th1-Th17 lymphocytes	4.25 (0.02–15.8)	0.16 (0.01–3.37)	.0001*

That the bold values indicates the data with statistical significance.

### 3.8 Clinical correlations

As previously specified in Methods and Materials, patients were divided considering spleen alterations, number of VOCs and number of transfusion procedures, afterwards a comparison between the outlined groups was performed. The significant results are reported below.

Regarding spleen alterations, we observed that patients who underwent surgical splenectomy or experienced autosplenectomy (10 patients, mean age 14.6 years, range 7–31 years), in comparison with patients with normal spleen function (43 patients, mean age 9 years, range 1–24 years), displayed an increase in CD4^+^ central memory T lymphocytes in absolute number (13.8 cells/mm^3^ vs. 0.4 cells/mm^3^, *p*-value ≤.05), an increase in CD19^+^ B lymphocytes in absolute number (126 cells/mm^3^ vs. 66 cells/mm^3^, *p*-value ≤.05) and a decrease in HLA-DR^low^ monocytes in percentage (1.6% vs. 6%, *p*-value ≤.05).

In the comparison between patients who underwent transfusion procedures with higher frequency (23 patients, mean age 8.7 years, range 1–22 years) and patients who underwent transfusion with lower frequency (30 patients, mean age 11.1 years, range 1–31 years), data showed an increase in CD19^+^ B lymphocytes in percentage (6.6% vs. 3.3%, *p*-value ≤.05) and an increase in CD35^+^ neutrophils (59172 MFI vs. 37927 MFI, *p*-value pì ≤.01) in patients with higher transfusion need.

Moreover, we observed that patients who experienced VOCs with higher frequency (16 patients, mean age 11.2 years, range 5–22 years) displayed a decrease in CD4^+^ RO + T cells in percentage (29.8% vs. 42.6%, *p*-value ≤.05), a decrease in CD4^+^ effector memory T cells in percentage (16.5% vs. 25.7%, *p*-value ≤.05) and absolute number (115/mm^3^ vs. 145/mm^3^, *p*-value ≤.05), an increase CD8^+^ TEMRA cells in percentage (24.3% vs. 13.3%, *p*-value ≤.05) in the comparison with patients who experienced VOCs with lower frequency (37 patients, mean age 9.6 years, range 1–31 years).

## 4 Discussion

Immune alterations associated with a proinflammatory pattern have been reported in SCD patients and actively contribute to the genesis of severe recurrent infections and vaso-occlusive complications, leading to a significant increase in morbidity and/or mortality (Chies and Nardi, 2001; Balandya et al., 2016; Williams and Thein, 2018).

In our study, we observed an increase in white blood cells in SCD patients mainly attributable to higher values of granulocytes and monocytes, as already described in literature (Allali et al., 2020). It is known that the increase in the absolute number of neutrophils correlates with the severity of the disease (Anyaegbu et al., 1998) and that SCD patient’s neutrophils show an activated pattern, with amplified adhesive properties in basal conditions, which increase during VOC (Fadlon et al., 1998, 64; Lum et al., 2004).

Moreover, the absolute number of monocytes is known to be increased in patients with SCD (Wongtong et al., 2015) with an activated profile (Belcher et al., 2000; Inwald et al., 2000; Wun et al., 2002).

This uncontrolled activation of innate immunity is a consequence of hemolysis through the release of heme and free Hb that act as DAMP and vaso-occlusion episodes with consequently repeated episodes of tissue ischemia and reperfusion that lead to a radical of oxygen damage and to the activation of innate and adaptive immune response (Kaul and Hebbel, 2000; Osarogiagbon et al., 2000; Belcher et al., 2003; 2005; Polanowska-Grabowska et al., 2010).

Therefore, even though our data are not fully definitive to draw this conclusion, we can speculate that this increase may be both a consequence of endothelial activation which stimulates the recruitment of granulocytes (Duits et al., 1996; Koehl et al., 2017) and a consequence of splenic alterations typical of SCD (Brousse et al., 2014).

Moreover, in SCD patients, we observed a reduced expression of CD62L (L-selectin). Diminished levels of L-selectin are associated with a shedding process that occurs during neutrophils activation (Bevilacqua and Nelson, 1993) and, since L-selectin interacts with the carbohydrates of the endothelial membrane, the reduced expression of CD62L can lead to a decrease in their marginal pool, thus contributing to hyperleukocytosis (Benkerrou et al., 2002). Therefore, we can hypothesize that this finding may be associated with an increased activation of neutrophils, even though we did not find significant alteration in other markers (CD16, CD11b and CD35) that can fully support our hypothesis.

We did not find differences in monocytes subpopulations (cM, iM and ncM) between patients and HD, but SCD cohort revealed a reduction in HLA-DR^low^ monocytes. These cells are part of a larger class of suppressive cells, called myeloid-derived suppressive cells (MDSC) having a crucial role as regulators of the transition from inflammatory state to immune suppression (Mengos et al., 2019). Thus, their reduction, leads to higher activation of the immune system (Kim et al., 2010).

Therefore, the activated pattern of neutrophils and monocytes in our cohort of patients may confirm the role of innate immunity in contributing to sterile inflammation in patients with SCD, since the very early stages of the disease in children.

Our data did not show differences in NK population between patients and HD. Abraham et al. (Abraham et al., 2019) showed that SCD patients without disease-modifying therapy had a higher number of NK cells with increased cytotoxicity, while NK cells from patients in therapy with HU did not show differences in number or phenotype respect to HD. Since 79% of our population was regularly taking hydroxyurea, our data is coherent with the aforementioned study.

On the other hand, we observed a reduction in iNKT cells in patients with SCD, this data disagree with other studies (Wallace and Linden, 2010), in which the expansion and activation of iNKT cells were observed in SCD patients compared to HD.

In this study, we observed a reduction in bone marrow hematopoietic precursors (CD34^+^ cells) in patients with SCD (in percentage and absolute numbers). Again, it can be hypothesized that this alteration could be a consequence of inflammation. In fact, Leonard et al. (Leonard et al., 2019) described an increase in inflammatory markers in the bone marrow (BM) of SCD compared with HD. Moreover, in the bone marrow of SCD patients there is an altered regulation of hematopoietic stem cell (HSC) homeostasis, due in part to hemolysis and oxidative stress (Tang et al., 2021). It is known that free heme can feed inflammatory process in SCD through the activation of TRL4 signaling (Belcher et al., 2014). In particular, Tang et al. (Tang et al., 2021) observed that TLR4/p65 activation can induce changing in the one marrow mesenchymal stromal cells ability to maintain hemopoietic stem cells in SCD, leading to their decrease. Therefore, inflammatory mechanism, through free heme and oxidative stress, may be associated with CD34^+^ cells reduction in SCD.

Our data did not highlight differences in total lymphocytes count and in CD19^+^ lymphocytes between patients and HD.

Unlike T cells, the number of B cells in SCD was generally found to be unaltered in other studies as well (Sanhadji et al., 1988; Wang et al., 1988), although occasional and modest increases in B cells have been described (Kaaba and al-Harbi, 1993; Nickel et al., 2015).

On the other hand, we observed a reduction in CD3^+^ cells in SCD patients with a reduced CD4^+^ count and an increase in CD8^+^ T cells.

In other studies (Belcher et al., 2003) the decrease in CD3^+^ T cells was mainly associated with a reduction in the CD4^+^ T cells count as a consequence of splenic alterations which can lead to a reduction of CD4+/CD8+ ratio (Sieber et al., 1985; Ferrante et al., 1987). Since only in a small proportion of patients we observed splenic alterations (7 out of 53 patients underwent surgical splenectomy and 3 of them experienced autosplenectomy), we can hypothesize that this increase may also be a consequence of a possible functional asplenia present in subjects without apparent splenic dysfunction. In fact, spleen is a frequent site of ischemic damage which progressively lead to fibrotic replacement of the parenchyma and consequent hyposplenism (El Hoss and Brousse, 2019).

To support this hypothesis, the comparison study between patients with and without splenic alterations resulted in just few statistical differences, thus observing a homogeneous distribution of immune system cells between the two populations.

We observed an increase in CD19^+^ B lymphocytes in patients who underwent surgical splenectomy. Since spleen is the site of functional maturation of B lymphocytes and since the majority of these cells resides within the spleen and the lymph nodes (LeBien and Tedder, 2008), splenic alteration may be associated with an increase in CD19^+^ cells, as already observed in other studies (Chelazzi et al., 1987).

Regarding CD4^+^ subpopulations, we observed an increase in central memory T cells, data consistent with other studies (Nickel et al., 2015). In other studies (Vingert et al., 2015), patients undergoing multiple transfusions showed an increased proportion of CD4^+^ central memory T cells, regardless their alloimmunization status. This data is in contrast with our study in which we observed no differences of central memory CD4^+^ cells in patients with higher transfusion need. On the other hand we observed that patients who experienced VOCs with higher frequency displayed a decrease in CD4^+^ memory and effectory memory T lymphocytes. Multicentric studies may be needed to properly understand the main trigger responsible of CD4^+^ memory T cells alterations also aimed at highlight differences according to specific clinical phenotypes (vaso-occlusive subphenotype, hemolysis and vasculopathy subphenotype, high HbF subphenotype, pain subphenotype) (Kato et al., 2018).

As for the increase in CD8^+^ naïve T cells, we think that this finding could be influenced by the small sample and the wide range age of enrolled patients.

Furthermore, we observed an increase in CD69^+^ CD4^+^ and CD8^+^ T cells. CD69 is an early activation marker which is expressed in the hematopoietic stem cells, T cells and many other cells of the immune system and it is also implicated in T cells differentiation and lymphocyte retention in lymphoid organ (Ziegler et al., 1994, 69). CD69 expression has been associated with both regulatory T cells and memory T cells and precursors (Ise et al., 2018).

In our population of patients with SCD we observed an increase in Treg CD39^+^ cells with a reduction in HLA-DR + T cells, data highlighted in other studies as well (Vingert et al., 2014). No differences were observed in the CD4^+^CD25^+^CD127 low subpopulation. Rêgo et al. (Rêgo et al., 2015) showed an increase in the frequency of regulatory T cells (especially CD4^+^CD25+FoxP3 cells) in SCD patients, whose in our population were not significant.

Higher levels of CD39^+^ T cells are associated with an overactivation of immunoregulatory functions of Tregs in SCD patients: in particular, the overexpression of CD39 leads to an increase in the production of adenosine and a decrease in the immune activation of T lymphocytes (Young et al., 2014; Allard et al., 2017).

Moreover, the expression of HLA-DR identifies the activated subpopulation of regulatory T cells with a role in suppressing the proliferation of conventional T lymphocytes *in vitro*, therefore, the low level of HLA-DR in Treg subpopulation may contribute to a higher immune activation (Baecher-Allan et al., 2006).

We can hypothesize that the increase in the regulatory T lymphocyte population could be linked to the body’s attempt to minimize the inflammation intrinsic to SCD (Vingert et al., 2014).

Regarding the T helper population, our data showed an increase in the Th2, Th1-17 and Th17 response. In vaso-occlusive crises, an increase in interleukin-4 (IL4) secretion was described, suggesting a shift in the response of CD4 + T cells towards a Th2 phenotype (Bundy et al., 2010).

Previous studies have also observed a predominant immunological alteration in alloimmunized SCD patients with a shift to a CD4^+^ T helper response toward Th2 and Th17 phenotypes, with increase production of Il-4 and Il-17 respectively (Bao et al., 2011).

Regarding chronic transfusions, as already highlighted, other studies showed that a higher transfusion rate can be associated with an increase in CD4^+^ central memory T lymphocytes and Treg population and these alterations may be associated with alloimmunization (Vingert et al., 2015). Moreover Nickel et al. demonstrated a significant increased percentage of CD41 T memory cells in alloimmunized SCD patients compared to non-alloimmunized patients (Nickel et al., 2015).

Despite the acute effect of increasing viscosity, chronic transfusion therapy seems to improve red blood cell deformability and aggregation with an increased tissue oxygenation (Detterich, 2018). However, other studies focused on the fact that the benefits of transfusion may vary depending on local flow rates, in fact large artery disease are prevented by chronic transfusion, while on the other hand the increased viscosity could worsen microvascular disease (Alexy et al., 2006). Moreover, recent evidences highlighted between splenectomy and vascular alteration, in particular thromboembolism and pulmonary arterial hypertension that may contribute to the alteration of the rheology of red blood cells in SCD (Bonderman et al., 2005; Crary and Buchanan, 2009). Therefore, it is utterly important to consider influence of transfusion and splenectomy in the changes of red blood cells as they are associated with hemolysis and vaso-occlusion that represent the main trigger for inflammatory processes (Wandersee and Hillery, 2016).

In particular, as already discussed, in our study we observed that patients with splenic alterations + displayed an increase in CD4^+^ central memory T lymphocytes and CD19^+^ B with a decrease in HLA-DR^low^ monocytes, while patients with higher transfusion need highlighted an increase in CD19^+^ B lymphocytes [maybe linked to alloimmunization (Hoffman et al., 2016)] and in CD35^+^ neutrophils [associated with an increased inflammatory response (Kobayashi et al., 2005)].

This study has some limitations. First, this is an observational, unicentric and retrospective study with small number of patients enrolled. Therefore, we believe that a multicenter study may be needed to increase the sample of the population to reach a better standardization of the data. Moreover, we observed significant differences regarding the age of our patients, that may have partially altered our results, considering the fluctuation of lymphocytes with age (Lin et al., 2016).

Moreover, further studies may be needed to assess whether specific therapy could change the immunological profile. Recently, Giulietti et al. (Giulietti et al., 2022) studied in-depth immunological alterations in SCD with many findings which were similar to the ones that we observed in SCD population (increase in WBC and neutrophils, decrease in CD3^+^ cells and CD4^+^ cells, increase in CD4^+^ central memory T cells), but, in addition, they observed immunological alteration probably consequent to the use of hydroxyurea. Therefore, it could be interesting to assess if recent therapies [such as Crizanlizumab (Ataga et al., 2017) or Voxelotor (Herity et al., 2021)] can lead to immunological changes as well.

In conclusion, we observed immunological alterations typical of an inflammatory status (increase in activated neutrophils and monocytes) associated with a peculiar Treg pattern (probably linked to a body attempt to minimize inflammation intrinsic to SCD).

Furthermore, our patients highlighted a T helper pathway possibly associated with alloimmunization in line with other studies, a decrease in iNKT cells and in CD34 cells when compared to HD.

Our data showed that immunological markers may have an important role in the understanding the pathophysiology of SCD.

In fact, we observed that our patients had immunologic alterations from a young age, so prospectively, multicenter studies may be needed to assess their implications on infectious risk and vaccine response. In addition, this study highlights how a thorough characterization of the immunophenotype in patients with SCD could be useful to better characterize their inflammatory status, with the ultimate goal of identifying patients most at risk for related chronic complications from an early age.

## Data Availability

The raw data supporting the conclusion of this article will be made available by the authors, without undue reservation.
